# Narrative review and creation of an institutional protocol for the use of fibrinolytics in parapneumonic effusion in children

**DOI:** 10.1016/j.jped.2025.01.005

**Published:** 2025-03-11

**Authors:** Flavia Garcia Frogeri, Andréa de Melo Alexandre Fraga, Fernando Augusto de Lima Marson, Antônio Gonçalves de Oliveira Filho, Márcio Lopes Miranda, Joaquim Murray Bustorff-Silva

**Affiliations:** a Universidade Estadual de Campinas (UNICAMP), Hospital de Clínicas, Disciplina de Cirurgia Pediátrica, Campinas, São Paulo, Brazil; b Universidade Estadual de Campinas (UNICAMP), Hospital de Clínicas, Departamento de Pediatria, Campinas, São Paulo, Brazil; c Universidade Estadual de Campinas (UNICAMP), Centro de Investigação em Pediatria (CIPED), Campinas, São Paulo, Brazil

**Keywords:** Children, Empyema, Pneumonia, Fibrinolytic agents

## Abstract

**Objective:**

Pneumonia is the leading cause of morbidity and mortality in children under 5 years old, with an increasing incidence of parapneumonic pleural effusion. Pleural effusion is a common complication, sometimes requiring surgical intervention. A literature review was conducted on parapneumonic pleural effusion and its treatment in the pediatric population, and an institutional protocol for intrapleural fibrinolysis was developed.

**Data sources:**

Articles from the past 15 years were reviewed in the databases PubMed-MEDLINE, LILACS, Cochrane, and Scielo using the terms pleural effusion, empyema, pneumonia, fibrinolytic, and children. A protocol for intrapleural fibrinolytic use in cases of parapneumonic pleural effusion was established.

**Summary of findings:**

Fifteen studies were included in the review. Chest ultrasound was the imaging modality used for diagnosis and monitoring. Most studies evaluated and compared the use of pleural drainage combined with fibrinolytics and video-assisted thoracoscopic surgery (VATS). The most used fibrinolytics were tissue plasminogen activator and urokinase. Hospitalization duration and adverse effects were similar across groups. The therapeutic failure rate of chemical debridement ranged from 0 to 37.2%. VATS and drainage combined with fibrinolytics were safe and well-tolerated, offering advantages over simple pleural drainage.

**Conclusions:**

Chemical debridement is cost-effective and less invasive, with complication rates and hospitalization times similar to VATS, making it preferable as a first-line treatment. The created protocol will standardize institutional practices and support evidence-based decision-making.

## Introduction

Community-acquired pneumonia (CAP) is the leading cause of morbidity and mortality in children aged 28 days to 5 years, and it usually occurs in healthy children, although it tends to be more severe in patients with comorbidities.^1^ Despite a decrease in pneumonia mortality over the past decade due to advances in medicine and the introduction of the pneumococcal vaccine, there has been an increase in the incidence of parapneumonic effusion, reaching rates of 0.6% to 2% among patients with pneumonia. The reasons for this increase are not fully understood, but it is believed that several factors play an important role, such as increased bacterial resistance, climate change, and the indiscriminate use of broad-spectrum antibiotics.^1^, ^2^, ^3^, ^4^, ^5^, ^6^, ^7^, ^8^, ^9^, ^10^, ^11^, ^12^, ^13^

The main causative agents of CAP are viruses, which rarely cause complicated pneumonia. Among bacteria, the most common etiological agent is Streptococcus pneumoniae, whose incidence has decreased following the introduction of the pneumococcal vaccine, yet it remains the primary bacterial cause of pneumonia. Other bacteria that can cause CAP include *Streptococcus pyogenes, Staphylococcus aureus, Haemophilus influenzae, Mycoplasma pneumoniae, Pseudomonas aeruginosa, and Mycobacterium tuberculosis*.^4^^,^^10^^,^^12^

Parapneumonic effusion is the most common complication of pneumonia and can be divided into three stages: Stage I - exudative, characterized by inflammatory and sterile fluid that typically resolves with antibiotic therapy; Stage II - fibrinopurulent, which begins when fibrin is deposited in the pleural space; and Stage III - organizing phase, during which a thick membrane forms over the visceral pleura, limiting lung expansion.^1^^,^^3^^,^^6^^,^^9^^,^^14^ The presence of parapneumonic effusion should be suspected in children who remain febrile or show no clinical improvement after 48–72 h of appropriate antibiotic therapy.^4^^,^^6^^,^^10^^,^^12^^,^^15^

The traditional treatment for empyema consists of antibiotic therapy and pleural drainage, which has a failure rate of up to 40% and often results in prolonged hospital stays, depending on the stage of the effusion, as this treatment does not allow adequate drainage of loculated areas. In such cases, surgical debridement using video-assisted thoracoscopic surgery (VATS) has been proposed as an alternative to avoid thoracotomy, followed by chemical debridement with the intrapleural instillation of fibrinolytic agents.^1^^,^^3^^,^^5^^,^^7^^,^^9^^,^^14^^,^^16^^,^^17^ The indications for either method vary significantly from one service to another and remain a subject of controversy in the relevant literature.

The objective of this study was to conduct a narrative review of the literature on parapneumonic effusion and its treatment in the pediatric population, with an emphasis on the use of intrapleural fibrinolytics, their indications and methods of use, as well as a comparison with the use of VATS. Based on this review, an institutional protocol for the use of intrapleural fibrinolysis was developed to standardize the management of complicated parapneumonic effusion cases in the Pediatric Surgery Department of the State University of Campinas (UNICAMP).

## Methods

The search for articles was conducted in the databases PubMed-MEDLINE, LILACS, SciELO, and Cochrane, using the following terms: empyema, pneumonia, fibrinolytic agents, and children. The study was submitted to and approved by the Research Ethics Committee (CAAE no. 76244123.4.0000.5404).

### Inclusion criteria

Studies published in the last 15 years, in Portuguese and English. This period was chosen because it coincides with the increased use of intrapleural fibrinolytics in the treatment of parapneumonic effusion. Clinical trials, simple literature reviews, non-randomized experimental studies, cohort studies, case-control studies, and observational studies were included. Only studies that evaluated the use of intrapleural fibrinolytics were selected.

### Exclusion criteria

Articles published more than 15 years ago, case reports, and studies published in other languages were excluded, as well as studies that did not assess the use of intrapleural fibrinolytics in the treatment of parapneumonic effusion.

### Data analysis from the literature

The data from the selected studies were organized into tables, and the results were analyzed descriptively. Based on the collected information, an institutional protocol was developed for managing cases of patients with complicated parapneumonic effusion (Stages II and III), aiming to specify the indications for the use of intrapleural fibrinolytics, as well as their method of use and follow-up.

## Results

Fifteen articles were selected, consisting of 9 retrospective cohorts, 3 randomized clinical trials, 2 prospective cohorts, and 1 national surveillance study and guideline creation. The most relevant information from each study (study type, participant sex, mean age, imaging exams used for diagnosis and follow-up, stages of pleural effusion, patient comorbidities, length of hospital stay, type of therapy, adverse effects related to therapy, treatment failure rates, and mortality) is presented in Tables 1 and 2.

**Table 1:** Demographic and diagnostic characteristics of participants included in the studies evaluated in the review.

Article	Study type	N	Mean age (years)	Male	Imaging exam	Stage of the effusion	Comorbidities
Segerer et al.^18^	National surveillance study	645	5	49%	US in 87%	I (40%), II (39%), III (8%)	38% (11% prematurity)
Nandan et al.^7^	Retrospective cohort	84	7.1	54.5%	US in 100%	II, III	Malnutrition in 78.5%
Gautam et al.^13^	Retrospective cohort	153	3.7	60%	US (71.2%), CT (45%)	II, III	-
Angurana et al.^19^	Retrospective cohort	205	66.4% < 5	70%	-	II, III	36% no imunization, 17.6% malnutrition, 15.6% viral infections
Van Loo et al.^17^	Retrospective cohort	60	4.7	57.1% (PDF), 60% (VATS/T)	US (74% PDF and 58% VATS/T) and CT (9% PDF and 8% VATS/T)	II, III	-
Oyetunji et al.^11^	Retrospective cohort	48	4.5	56%	US and CT	II, III	-
Marhuenda et al.^12^	Randomized clinical trial	103	4.6 (PDF), 4.1 (VATS/T)	59.2%	US and CT	II, III	-
Livingstone, Colozza et al.^5^	Retrospective cohort	67	6.1 (PDF) 5.2 (VATS/T)	38% (PDF), 46% (VATS/T)	US	II	-
Griffith et al.^6^	Retrospective cohort	115	4.9	47.8%	US in 82.6% and CT in 1.7%	II, III	13.9% (25% asthma)
Cobanoglu et al.^14^	Randomized clinical trial	54	7.3±2.76 (PDF), 8.7±2.6 (VATS/T)	59.2%	US and CT	II, III	20.3%
Baram et al.^9^	Prospective cohort	95	6.3	47.4%	-	II, III	-
Rodriguez et al.^1^	Retrospective cohort	35	4	51.4%	XR and US	II, III	-
Peter et al.^16^	Randomized clinical trial	36	5.2±4.2 (PDF), 4.8±4.3 (VATS/T)	-	US and CT	II, III	-
Livingstone et al.^20^	Retrospective cohort	314	5.3	50%	XR and US	I (9%), II and III (91%)	9% (asthma)
Grasior et al.^21^	Prospective cohort	102	5.8±4.6 (PDF), 7.7±4.9 (VATS/T)	58% (PDF), 24% (PDF+VATS)	-	II, III	-

PDF, Pleural drainage + fibrinolytic; VATS/T, VATS or Thoracotomy; T, Thoracotomy; XR, Chest X-ray; US, Thoracic ultrasound; CT, Chest computed tomography; N, number of participants; -, No data or doesn't apply; %, percentage.

**Table 2:** Therapeutics instituted in participants included in the studies evaluated in the review.

Article	Treatment (%)			Length of hospital stay (days)			Fibrinolytic	Adverse event	Therapeutic failure rate (%)			Mortality
	PD	PDF	VATS/T	PD	PDF	VATS/T			PD	PDF	VATS/T	
Segerer et al.	24	14	7	17	16	17	-	-	-	0	0	-
Nandan et al.	52.3	47.6	0	24.32±10.18	17.51±4.57	-	Urokinase	None	20.4	10	-	0
Gantam et al.	15.2	15.9	19.9 (VATS), 46.4 (T)	14	14	11.3	-	-	-	-	-	-
Angurana et al.	36	33.7	27.8	17.2±6.3			Streptokinase	-	28.2	29	0	3.9
Van Loo et al.	0	58.33	41.47	-	13.54±6.24	16.48±8.17	Urokinase	None	-	14.3	-	-
Oyetunji et al.	0	100	0	-	8	-	tPA	-	-	4.2	-	0
Marhuenda et al.	0	48.5	51.5	-	13	14	Urokinase	18.9 (VATS), 18 (T)	-	10	15.1	0
Livingstone, Colozza et al.	0	58	42	-	9	8	tPA	1	-	13	4	0
Griffith et al.	0	74.8	16.5 (VATS), 8.7 (T)	-	7.5		Urokinase	8.2	-	37.2	-	0
Cobanoglu et al.	0	50	50	-	10.37±2.29	7.41±1.45	Streptokinase	12.96	-	29.63	22.23	0
Baram et al.	0	100	0	-	7.3	-	tPA	-	-	1.1	-	-
Rodriguez et al.	0	28.57	71.43	-	13	15	Urokinase	2.8	-	29	16	0
Peter et al.	0	50	50	-	6.8±2.9	6.9±3.7	tPA	None	-	16.6	0	0
Livingstone et al.	0	100	0	-	11	-	tPA	-	-	34	-	0
Grasior et al.	0	100	0	-	7.2±3.2	-	tPA	None	-	15.7	-	0

PD, Pleural drainage; PDF, Pleural drainage + fibrinolytic; VATS/T; VATS or Thoracotomy; T, Thoracotomy; tPA, Tissue plasminogen activator; US, Thoracic ultrasound; CT, Chest computed tomography; -, No data or doesn't apply; %, percentage.

The number of patients evaluated in each study ranged from 35 to 645, and the mean age varied from 3.7 to 8.7 years. The male sex was predominant in 9 studies.^1^^,^^2^^,^^3^^,^^7^^,^^11^^,^^13^^,^^14^^,^^17^, ^18^, ^19^ The imaging exam most used for the diagnosis and follow-up of parapneumonic pleural effusion was chest ultrasound, while computed tomography of the chest was used to evaluate complex cases, with suspected lung abscess or bronchopleural fistula. The stages of pleural effusions were predominantly II and III, with only 2 studies including effusions at stage I.^18^^,^^20^ The categorization of the effusion was primarily based on ultrasound characteristics, defined as stage I for a fluid effusion, stage II for an effusion with loculations and septations, and stage III when thickening of the visceral pleura was identified, already showing suggestive signs of pulmonary entrapment. Only 6 studies provided information on the comorbidities of the patients,^6^^,^^7^^,^^14^^,^^18^, ^19^, ^20^ with the most frequent being malnutrition, asthma, and prematurity.

Regarding the established therapy, most studies evaluated and compared the use of pleural drainage associated with intrapleural fibrinolytics and VATS, with only 4 studies also including patients who underwent isolated pleural drainage.^7^^,^^13^^,^^18^^,^^19^ All patients received antibiotic therapy, with varying treatment durations, which were not specified in most studies. The most used fibrinolytics were tissue plasminogen activator (tPA)^2^^,^^5^^,^^9^^,^^11^^,^^16^^,^^20^ and urokinase.^1^^,^^3^^,^^6^^,^^7^^,^^17^ Streptokinase was used in only 2 studies.^14^^,^^19^ The type of fibrinolytic used was not specified in 2 studies.^13^^,^^18^

The method of using the fibrinolytics was uniform among studies considering each substance used. Streptokinase was administered as a solution of 250,000 U/100 mL in saline, with an infused volume of 70-120 mL per application, once a day, keeping the drain clamped for 4-6 hours after infusion, for 3-5 consecutive days. Drains were maintained on continuous suction with pressures between -15 to -20 cm H_2_O.^14^ Urokinase was administered in two ways. The first consisted of a dose of 10,000 UI/kg/day for 3 days, diluted to 1000 UI/mL^1^. The second used 40,000 UI of urokinase diluted in 40 mL of saline, every 12 hours for 3 days for those over one year of age, and 20,000 UI diluted in 20 mL of saline for those under one year, with the drain kept closed for 4 hours after the instillation of the fibrinolytic. Some studies maintained the drains in continuous suction.^3^^,^^6^^,^^7^^,^^17^ tPA was also administered in two different ways, the first being used by most studies, with a dosage of 4 mg of tPA diluted in 20-40 mL of saline, maintaining the drain clamped for 1 hour and starting continuous suction afterward at -20 cmH2O, once a day, for 3 consecutive days.^5^^,^^11^^,^^16^^,^^20^ The second method of administering alteplase, used in one study, was a dosage of 0.1 mg/kg/dose diluted in 10-30 mL of saline, also given once a day for 3 consecutive days, with the drain kept clamped for one hour.^9^

Chest drains were removed according to the clinical status of the patients and the drainage output. Angurana et al. (2019) established a drainage output of < 10-15 mL/day,^19^ Oyetunji et al. (2020) and Gasior et al. (2013) < 1 mL/kg/day,^2^^,^^11^ and Rodriguez et al. (2022) < 20–40 mL/day.^1^

The length of hospital stay did not differ between the groups undergoing VATS and pleural drainage associated with intrapleural fibrinolytics, except for the study by Cobanoglu et al. (2011), which identified a shorter hospital stay in the VATS group (7.41±1.45 vs. 10.37± 2.29).^14^

Regarding the adverse effects of therapies, 4 studies reported no complications,^7^^,^^16^^,^^17^^,^^21^ 6 studies did not report these data,^9^^,^^11^^,^^13^^,^^18^, ^19^, ^20^ and in the remaining studies, the incidence of side effects varied from 1-18.9%.^1^^,^^3^^,^^5^^,^^6^^,^^14^ Identified complications included chest pain, fever, tachycardia, bleeding, aforia, bronchopleural fistula, and bronchospasm. Two studies compared complication rates post-VATS and post-pleural drainage associated with intrapleural fibrinolytics, finding no significant differences.^3^^,^^8^ One of them was a meta-analysis that found no difference between the incidence of adverse events (RR = 0.6 [95% CI = 0.3–1.2]) but identified a lower need for reintervention in the VATS group (RR = 0.55 [95% CI = 0.34–0.88]).^8^

The rate of therapeutic failure for chemical debridement (pleural drainage associated with intrapleural fibrinolytics) varied from 0 to 37.2% and was not quantified in one study.^13^ After the failure of chemical debridement, VATS was mostly used as a rescue therapeutic option. The failure rate for VATS varied from 0 to 22.2% and was not quantified in nine studies.^2^^,^^6^^,^^7^^,^^9^^,^^11^^,^^13^^,^^17^^,^^19^^,^^20^

The mortality rate, analyzed in 15 articles, varied from 0 to 3.9%. Four articles did not assess mortality.^9^^,^^13^^,^^17^^,^^18^

## Discussion

The results of this review showed that the treatment of parapneumonic pleural effusion in children varies from publication to publication, with some controversies regarding the best way to evaluate and treat this complication of pneumonia.

From a clinical perspective, the diagnosis of parapneumonic effusion should be suspected when there is no improvement or there is the clinical deterioration of the patient despite appropriate antibiotic therapy for at least 48 hours and can be confirmed by a chest X-ray. According to the guidelines from the Outcomes and Clinical Trials Committee of the American Pediatric Surgery Association (APSA), chest ultrasound is the best imaging study to assess the pleural space in children, as it is more sensitive than X-ray for detecting small effusions and can evaluate septations and differentiate effusions from pulmonary consolidations, and it should be used to establish the stage of pleural effusion. Computed tomography of the chest, in addition to exposing patients to radiation and potentially increasing the long-term cancer risk, does not provide additional information to ultrasound and should only be performed in cases of diagnostic uncertainty or in complex cases when there is suspicion of lung abscess or bronchopleural fistula.^2^^,^^4^^,^^6^^,^^10^^,^^12^^,^^15^^,^^22^

The treatment of pneumonia complicated by pleural effusion consists of clinical support and antibiotic therapy, which may or may not be associated with interventional procedures. Antibiotic treatment is usually effective in patients with small effusions, without mediastinal shift or respiratory compromise, and the choice of antibiotic should take into account local antibiotic resistance patterns and the child's comorbidities.^10^ Supportive treatment includes oxygen supplementation if needed, respiratory physiotherapy, adequate nutrition, and correction of electrolyte disturbances, with many patients often requiring intensive care treatment.^4^^,^^6^^,^^10^^,^^12^^,^^15^^,^^18^^,^^23^, ^24^, ^25^

One of the procedures that can be useful in cases of pneumonia complicated by pleural effusion is diagnostic and therapeutic thoracentesis, which has been less utilized in pediatric patients because multiple thoracenteses are usually necessary, reducing its advantage over pleural drainage. Therapeutic alternatives include simple pleural drainage or intrapleural instillation of fibrinolytic agents and VATS. These interventions are usually performed by pediatric surgeons and are typically necessary in cases of symptomatic pleural effusions, loculated effusions, or moderate to large volume effusions. The protocol proposed by APSA indicates pleural drainage for large effusions (> 2 cm thickness on X-ray in the supine position), loculated effusions, and symptomatic moderate effusions (1–2 cm), or when there is clinical deterioration despite appropriate treatment. Additionally, it is recommended that small drains (less than 14 Fr) be used whenever possible, as they are better tolerated, cause less discomfort for the patient, and have the same efficacy as the thicker drains.^2^^,^^26^

The use of intrapleural fibrinolytics aims to facilitate more effective drainage of infected fluid by acting on the pathophysiology of empyema formation, as infected pleural space leads to fibrin deposition and reduced activity of fibrinolytics, forming septations and loculations that are dissolved by external fibrinolytic agents.^2^^,^^4^^,^^15^^,^^18^^,^^26^^,^^27^ The first fibrinolytic used for empyema treatment was streptokinase; however, the risk of delayed hypersensitivity reaction led to its replacement by urokinase, a very effective fibrinolytic, however not available in many hospitals in Brazil. tPA emerged in 2000 as an alternative to urokinase.^8^^,^^9^^,^^28^

The success rate associated with fibrinolytic treatment in the consulted literature varied from 62.8% to 98.9%. One study^20^ aimed at evaluating predictors of treatment failure in children with empyema treated with drainage associated with fibrinolytics indicated that early admission to the intensive care unit and the presence of positive blood cultures were associated with a higher likelihood of treatment failure (53% vs. 28% if these factors were absent).

VATS can be used as a first treatment option for complicated pleural effusions or as an alternative after failure of chemical debridement, which is diagnosed when there is no clinical improvement, insufficient drainage of pleural fluid, and persistence of empyema in imaging studies.^1^

Regarding complications of chemical and mechanical fibrinolysis, a meta-analysis evaluated a total of 1654 procedures (81% VATS and 19% drainage associated with fibrinolytic) and identified that the most common complication associated with VATS was persistent bronchopleural fistula, while in patients undergoing chemical debridement, the complications were chest pain and change in drain position, with only one patient in the studies experiencing bleeding after tPA.^8^ A randomized clinical trial found a complication incidence of 12.8% in the group undergoing chemical debridement, with the main complications being hypertension, hemorrhage, chest pain, and aforia, and a complication rate of 11.1% in the VATS group, with the main complications being prolonged air leak and surgical site infection.^14^

The data collected in this literature review suggest that both VATS and drainage associated with fibrinolytics are safe, well tolerated, and have advantages over simple pleural drainage in cases of complicated effusions (stages II and III).^8^^,^^17^ The use of intrapleural fibrinolytics was cheaper in 3 studies evaluated by Pacilli et al. (2019) in their systematic review, which was also noted by Peter et al. (2019), who showed a cost of $7.600 ± $5.400 for drainage associated with fibrinolytics and $11.700 ± $2.900 for VATS.^8^^,^^16^ In the study by Cobanoglu et al. (2011), fibrinolytics also had a lower cost ($386,672 ± $72,060 vs. $957,487 ± $137,238).^14^

Considering that chemical debridement has a lower cost and is a less invasive procedure that can be performed without the need for general anesthesia, the service has opted to use it as a first-line treatment for complicated effusions, reserving VATS for cases of failure of fibrinolytic use or in situations where the use of fibrinolytics is contraindicated (bleeding or evident bronchopleural fistula at diagnosis).^2^^,^^29^

### Protocol for the treatment of parapneumonic pleural effusion

Based on a literature review and with the aim of standardizing practices, a protocol for managing complicated parapneumonic pleural effusion (stages II and III) has been created by the Pediatric Surgery Division of the Hospital de Clínicas at Unicamp. The diagnosis is based on clinical examination and chest X-ray in the posteroanterior and lateral views. It has been established that the imaging method for staging the effusion is thoracic ultrasound, which should preferably be performed by the radiology team using a linear probe positioned perpendicularly to the patient's chest and moved either perpendicular or parallel to the ribs. The evaluation should be performed systematically: the chest should be divided into three quadrants—anterior, lateral, and posterior—defined by the parasternal line, anterior axillary line, and posterior axillary line, with the thoracic cavity visualized down to the diaphragm. In cases where a simple pleural effusion (stage I) with thickness > 2 cm is identified, thoracentesis and possible chest drainage should be performed (depending on the macroscopic characteristics of the effusion).^30^^,^^31^ If ultrasound identifies a simple effusion with a maximum thickness of 1-2 cm, the indication for thoracentesis, chest drainage, or isolated antibiotic therapy will depend on the clinical conditions of the child, being indicated for patients who do not respond adequately to antibiotic therapy or who have persistent fever and poor ventilatory patterns.

When a complicated pleural effusion is identified by ultrasound, with thickness > 2 cm or between 1-2 cm in a clinically deteriorating patient, the first-line treatment is pleural drainage with intrapleural instillation of a fibrinolytic agent. Initially, the preferred drain, in the absence of associated pneumothorax, is the pig-tail drain, as its diameter is smaller and it can be placed at the bedside without the need for general anesthesia.

The fibrinolytic agent of choice is tPA (Alteplase), as it is available at the institution, has safety documented in the literature, and poses a lower risk of hypersensitivity reactions compared to streptokinase. The method of using tPA was chosen based on literature: 4 mg of tPA diluted in 20-40 mL of saline solution and instilled through the pleural drain, keeping the drain clamped for 1 hour and then maintaining the drain in water seal, with rigorous quantification of the output. This procedure should be initiated immediately after pleural drainage and can be repeated for two consecutive days, totaling three doses.

Contraindications for the use of tPA include suspected bronchopleural fistula and blood dyscrasias. During treatment, attention should be paid to adverse effects such as bleeding, chest pain, and dyspnea. If the patient remains febrile, shows clinical deterioration, and has unsatisfactory drainage output after the three doses of intrapleural Alteplase, suspicion of persistent pleural collection should arise, and a chest X-ray should be performed. If there is suspicion of pleural effusion persistence on the X-ray, a new thoracic ultrasound should be carried out for a better assessment of the pleural space. If the hypothesis of maintained pleural collection is confirmed, the possibility of VATS (Video-Assisted Thoracoscopic Surgery) as a rescue therapy may be considered.

**Figure 1:**
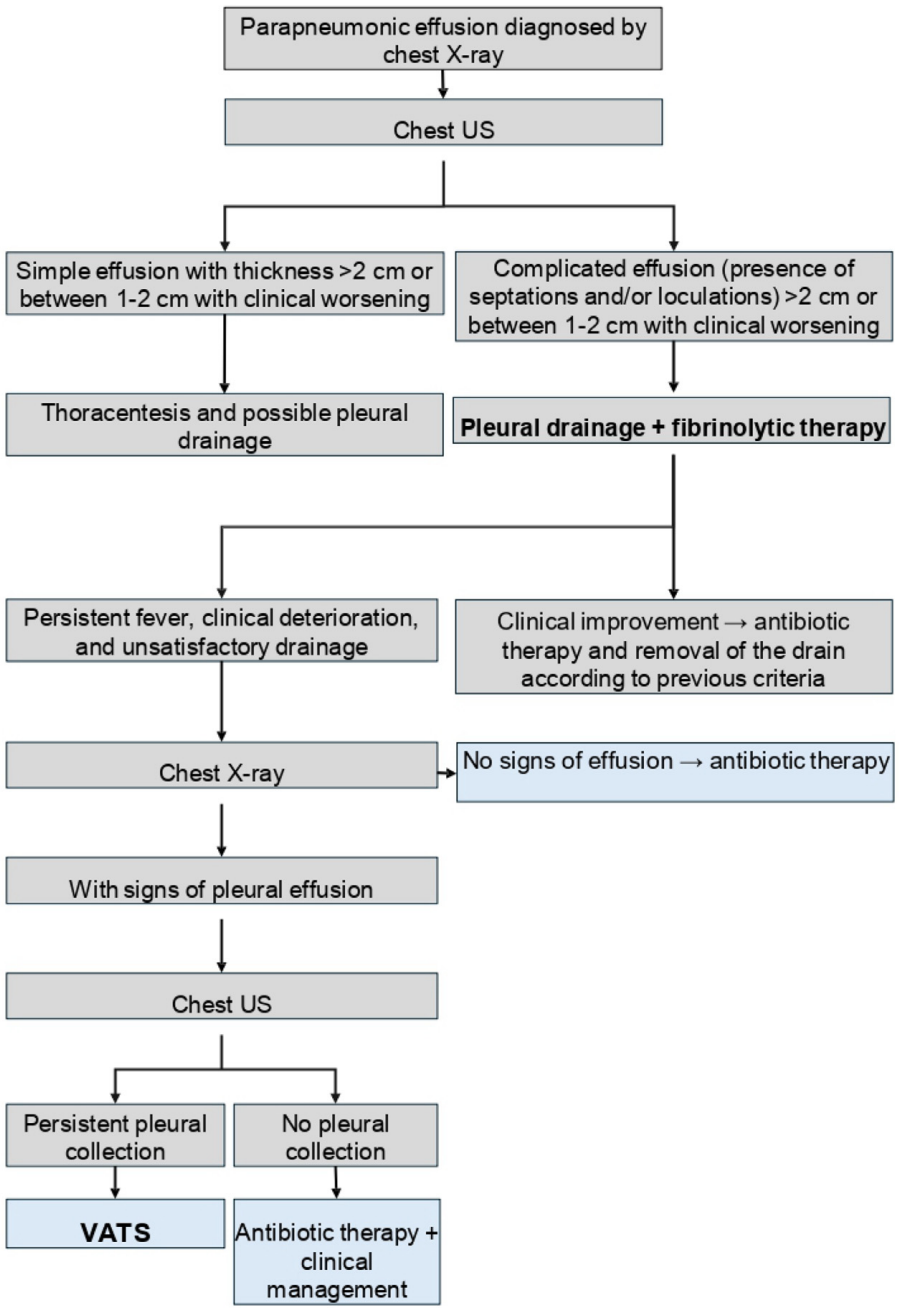
Patient management protocol.

In patients with good progress, the criteria for drain removal are: good clinical condition, normal body temperature over the past 48 hours, chest X-ray showing no signs of pleural effusion, and pleural drainage output < 1 mL/kg/day. A graphical representation of the protocol is presented in Figure 1.

## Conclusion

The literature review conducted allows us to conclude that pleural drainage associated with intrapleural instillation of fibrinolytic agents constitutes a safe and effective option for the treatment of patients with complicated parapneumonic effusion. Based on these conclusions, a protocol was created to standardize the institution's practices and facilitate evidence-based decision-making aimed at safe, effective, and minimally invasive therapy.

## Conflicts of interest

The authors declare no conflicts of interest.

## References

[1] Rodriguez M.R., Perez J.I., Ruenda F.V., Pascual F.J., Torres S.R., Esteban RM. (2022). Fibrinolysis versus thoracoscopy: Comparison of results in empyema management in the child. Ann Thorac Med.

[2] Islam S., Calkins C.M., Goldin A.B., Chen C., Downard C.D., Huang E.Y. (2012). The diagnosis and management of empyema in children: a comprehensive review from the APSA outcomes and Clinical Trials Committee. J Pediatr Surg.

[3] Marhuenda C., Barcelo C., Fuentes I., Guillén G., Cano I., López M. (2014). Urokinase versus VATS for treatment of empyema: a randomized multicenter clinical trial. Pediatrics.

[4] Erlichman I., Breuer O., Shoseyov D., Cohen-Cymberknoh M., Koplewitz B., Averbuch D. (2016). Complicated community acquired pneumonia in childhood: Different types, clinical course, and outcome. Pediatr Pulmonol.

[5] Livingstone M.H., Colozza S., Vogt K.N., Merritt N., Butter A. (2016). Making the transition from video assisted thoracoscopic surgery to chest tube with fibrinolytics for empyema in children: any change in outcomes?. Can J Surg.

[6] Griffith D., Boal M., Rogers T. (2018). Evolution of practice in the management of parapneumonic effusion and empyema in children. J Pediatr Surg.

[7] Nandan D., Agarwal S., Bidhuri N., Shrivastava K., Nanda P., Lata S. (2019). Role of Intrapleural urokinase in empyema thoracis. Indian J Pediatr.

[8] Pacilli M., Nataraja RM. (2019). Management of paediatric empyema by video-assisted thoracoscopic surgery (VATS) versus chest drain with fibrinolysis: Systematic review and meta-analysis. Paediatr Respir Rev.

[9] Baram A., Yaldo F. (2020). Pediatric thoracic empyema—outcomes of intrapleural thrombolytics: ten years of experience. Glob Pediatr Health.

[10] Benedictis F.M., Kerem E., Chang A.B., Colin A.A., Zar H.J., Bush A. (2020). Complicated pneumonia in children. Lancet.

[11] Oyetunji T.A., Dorman R.M., Svetanoff W.J., Depala K., Jain S., Dekonenko C. (2020). Declining frequency of thoracoscopic decortication for empyema — redefining failure after fibrinolysis. J Pediatr Surg.

[12] Masarweh K., Gur M., Toukan Y., Bar-Yoseph R., Kassis I., Gut G. (2021). Factors associated with complicated pneumonia in children. Pediatr Pulmonol.

[13] Gautam A., Wiseman G., Legg R., Lindsay D., Puvvadi R., Rathnamma B.M. (2022). Management of pediatric thoracic empyema in the North Queensland Region of Australia and impact of a local evidence-based treatment guideline. Pediatr Infect Dis J.

[14] Cobanoglu U., Sayir F., Bilici S., Melek M. (2011). Comparison of the methods of fibrinolysis by tube thoracostomy and thoracoscopic decortication in children with stage II and III empyema: a prospective randomized study. Pediatr Rep.

[15] James C.A., Braswell L.E., Pezeshkmehr A.H., Roberson P.K., Parks J.A., Moore MB. (2016). Stratifying fibrinolytic dosing in pediatric parapneumonic effusion based on ultrasound grade correlation. Pediatr Radiol.

[16] St Peter S.D., Tsao K., Spilde T.L., Keckler S.J., Harrison C., Jackson M.A. (2009). Thoracoscopic decortication vs tube thoracostomy with fibrinolysis for empyema in children: a prospective, randomized trial. J Pediatr Surg.

[17] van Loo A., van Loo E., Selvadurai H., Cooper P., Asperen P.V., Fitzgerald DA. (2014). Intrapleural urokinase versus surgical management of childhood empyema. J Paediatr Child Health.

[18] Segerer F.J., Seeger K., Maier A., Hagemann C., Schoen C. (2017). Therapy of 645 children with parapneumonic effusion and empyema—a German nationwide surveillance study. Pediatr Pulmonol.

[19] Angurana S.K., Kumar R., Singh M., Verma S., Samujh R., Singhi S. (2019). Pediatric empyema thoracis: what has changed over a decade?. J Trop Pediatr.

[20] Livingstone M.H., Cohen E., Giglia L., Pirrello D., Mistry N., Mahant S. (2016). Are some children with empyema at risk for treatment failure with fibrinolytics? A multicenter cohort study. J Pediatr Surg.

[21] Gasior A.C., Knott E.M., Sharp S.W., Ostlie D.J. (2013). Holcomb 3^rd^ GW, St Peter SD. Experience with an evidence-based protocol using fibrinolysis as first line treatment for empyema in children. J Pediatr Surg.

[22] Yilmaz H.L., Özkaya A.K., Sarı Gökay S., Tolu Kendir Ö, Şenol H. (2017). Point-of-care lung ultrasound in children with community acquired pneumonia. Am J Emerg Med.

[23] Knebel R., Fraga J.C., Amantea S.L., Isolan PB. (2017). Videothoracoscopic surgery before and after chest tube drainage for children with complicated parapneumonic effusion. J Pediatr.

[24] Freitas S., Fraga J.C., Canani F. (2009). Thoracoscopy in children with complicated parapneumonic pleural effusion at the fibrinopurulent stage: a multi-institutional study. J Brasil Pneumol.

[25] Schneider C.R., Gauderer M.W., Blackhurst D., Chandler J.C., Abrams RS. (2010). Video-assisted thoracoscopic surgery as a primary intervention in pediatric parapneumonic effusion and empyema. Am Surg.

[26] Andrés-Martín A., Escribano Montaner A., Figuerola Mulet J., García García M.L., Korta Murua J., Moreno-Pérez D. (2020). Consensus document on community-acquired pneumonia in children. SENP-SEPAR-SEIP. Arch Bronconeumol (Engl Ed).

[27] Weinstein M., Restrepo R., Chait P.G., Connolly B., Temple M., Macarthur C. (2004). Effectiveness and safety of tissue plasminogen activator in the management of complicated parapneumonic effusions. Pediatrics.

[28] Nie W., Liu Y., Ye J., Shi L., Shao F., Ying K., Zhang R. (2014). Efficacy of intrapleural instillation of fibrinolytics for treating pleural empyema and parapneumonic effusion: a meta-analysis of randomized control trials. Clin Respir J.

[29] Tsao K., St Peter S.D., Sharp S.W., Nair A., Andrews W.S., Sharp R.J. (2008). Current application of thoracoscopy in children. J Laparoendosc Adv Surg Tech A.

[30] Claes A.S., Clapuyt P., Menten R., Michoux N., Dumitriu D. (2017). Performance of chest ultrasound in pediatric pneumonia. Eur J Radiol.

[31] Bobillo-Perez S., Girona-Alarcon M., Jordan I., Gargallo MB. (2020). Lung ultrasound in children: what does it give us?. Paed Resp Rev.

